# Institutional Educational and Support Intervention in Breastfeeding in a Postnatal Unit in Spain: A Quasi-Experimental Pre-Post Study

**DOI:** 10.3390/nu18213702

**Published:** 2026-11-08

**Authors:** Pedro Hidalgo-Lopezosa, Patricia Ruz-Cea, Laura Moreno-Domínguez, Laetitia Emmanuelle Crosse, Cristina Quirós-Córdoba, Rubén García-Fernández, Pablo Jesús López-Soto, Inmaculada Ortiz-Esquinas

**Affiliations:** 1 Instituto Maimónides de Investigación Biomédica de Córdoba (IMIBIC), Facultad de Medicina, Enfermería y Fisioterapia, Universidad de Córdoba, 14004 Córdoba, Spain; n82losop@uco.es (P.J.L.-S.); inmaculada.ortiz.esquinas.sspa@juntadeandalucia.es (I.O.-E.); 2 Hospital Universitario Reina Sofía de Córdoba, 14004 Córdoba, Spain; moreno.dominguez.sspa@juntadeandalucia.es (L.M.-D.); laetitia.crosse.sspa@juntadeandalucia.es (L.E.C.); cristina.quiros.sspa@juntadeandalucia.es (C.Q.-C.); 3 SALBIS Research Group, Facultad de Ciencias de la Salud, Campus de Ponferrada, Universidad de León, 24401 León, Spain; rgarcf@unileon.es

**Keywords:** breastfeeding, midwife, nursing, intervention

## Abstract

**Background/Objectives**: There is international consensus that breastfeeding is the best form of nutrition for newborns. Many studies demonstrate the effectiveness of educational interventions in maintaining exclusive breastfeeding up to 6 months. The aim was to assess the effectiveness of an educational and support intervention in breastfeeding by a lead midwife on the postnatal ward. **Methods**: A retrospective quasi-experimental pre-post study complemented by a supplementary descriptive survey was conducted at a tertiary hospital in southern Spain with women (*N* = 136) who expressed a desire to breastfeed their babies. An institutional intervention, consisting of the support provided by a midwife employed exclusively to offer support with breastfeeding for a period of two months, was carried out. An inferential analysis was performed using the corresponding statistics. A multiple logistic regression analysis was performed to identify potential confounding variables and determine factors associated with the dependent variable of exclusive breastfeeding at 6 months. In addition, a brief institutional survey was conducted among these women after the first 6 months postpartum as a complementary component to the quantitative analysis. Data were obtained from hospital records and from the survey. **Results**: Exclusive breastfeeding at discharge and at two months postpartum was higher in the intervention group than in the control group. The need for supplementary feeding and phototherapy among infants was lower in the intervention group. A higher level of satisfaction with the support received regarding breastfeeding was also observed among mothers in the intervention group (95.7% vs. 63%; *p* < 0.001). However, we did not detect a between-group difference in exclusive breastfeeding at six months. **Conclusions**: Availability of support from a breastfeeding specialist midwife in hospital and in the weeks following birth can assist women to exclusively breastfeeding and supports to improve maternal satisfaction.

## 1. Introduction

The World Health Organization (WHO) and the United Nations Children’s Fund (UNICEF) recommend that infants be exclusively breastfed for the first six months of life due to its many benefits (strengthening the immune system, reducing chronic diseases, strengthening emotional bonds, cognitive development, etc.) [1]. The WHO defines exclusive breastfeeding as when ‘an infant receives only breast milk, no other liquids or solids are given—not even water, with the exception of oral rehydration solution, or drops/syrups of vitamins, minerals or medicines’ [2]. Exclusive breastfeeding for the first 6 months is beneficial to the neurodevelopment of infants, especially in those with severe neonatal jaundice [3]. After six months, breastfeeding should continue as an important component of the baby’s nutrition until the age of two [1,4]. The Initiative on Humanization of Birth Care and Breastfeeding promoted by WHO and UNICEF has contributed to improved breastfeeding outcomes [5].

There are numerous factors that promote breastfeeding, such as the mother’s level of university education, older maternal age, or a full-term birth; conversely, maternal smoking is associated with a lower likelihood of breastfeeding [6]. According to various studies, a common reason for discontinuing breastfeeding is the mother’s mistaken perception that the newborn is not feeding well during the first few weeks of life, which explains why the percentage of exclusive breastfeeding is declining [7]. Mothers’ preconceptions can hinder the successful practice of exclusive breastfeeding [8].

These breastfeeding problems can be addressed through effective educational and support programmes that can improve knowledge of breastfeeding as early as in antenatal care, or through postnatal interventions [8,9]. In this regard, many studies demonstrate the effectiveness of these educational interventions in maintaining exclusive breastfeeding up to 6 months [8,9,10,11]. A meta-analysis incorporating 63 studies found that breastfeeding counselling is an effective public health intervention for increasing rates of breastfeeding and exclusive breastfeeding [12].

Support from midwives/nurses and the presence of a midwife/nurse responsible for breastfeeding and monitoring mothers also appears to be associated with better outcomes in terms of longer duration of breastfeeding [13]. Furthermore, the authors associate a longer duration of breastfeeding with greater mental well-being in mothers [13]. Another Cochrane review showed that remote support and follow-up, as well as educational interventions on breastfeeding, significantly improve exclusive breastfeeding, attitudes towards breastfeeding and knowledge about breastfeeding [14].

In Spain, around 80–90% of mothers wish to breastfeed and do so after giving birth. This percentage is high and reflects a positive initial attitude towards breastfeeding. According to the latest data from the 2023 Spanish Health Survey (ESdE 2023), the rate of exclusive breastfeeding at 6 months was 47%, the highest figure in the entire historical series and an increase of 8 percentage points compared with the data collected in 2017 [15]. This figure brings Spain closer to the target set by the World Health Organization (WHO), which advocates increasing the rate of exclusive breastfeeding in the first 6 months of life to 60% [15].

The aim of this study was to assess the effectiveness of an educational and breastfeeding support intervention carried out by a midwife during the postnatal ward stay in terms of exclusive breastfeeding rates.

## 2. Materials and Methods

### 2.1. Design, Population and Setting of the Study

An analytical observational study with a retrospective pre-post quasi-experimental design, complemented by a supplementary descriptive survey, was conducted. The study took advantage of the temporary implementation of an institutional intervention in which a referral midwife was employed to support breastfeeding in the postpartum unit. The study was conducted with puerperal women who expressed their desire to exclusively breastfeed their infants in a tertiary hospital in the south of Spain, using data from the medical records of women who gave birth in this hospital. The inclusion criteria were women of legal age (over 18) who had given birth at the Hospital, who breastfed their infants, and whose newborns had not been admitted to the neonatology unit. Given the institutional focus on improving support among women actively breastfeeding during admission, our primary analysis targeted dyads who breastfed during hospitalization, recognizing this as a service evaluation of support quality within breastfeeding dyads.

The institutional intervention was non-randomized and consisted of the presence of a breastfeeding referral midwife whose role was to advise, support, resolve queries, and provide care about breastfeeding for a period of two months in the postpartum ward. This activity was carried out with all the women who expressed a desire to breastfeed their infants. After giving birth, all women are asked whether they intend to breastfeed their babies, and those who agree begin early breastfeeding in the delivery room. The decision not to breastfeed was respected at all times as just another option. The intervention group included all women (*n* = 68) who received the educational/care intervention by the breastfeeding referral midwife, plus routine care. The sample consisted of all the women except those who did not meet the inclusion criteria, while the control group consisted of women (*n* = 68) who received the normal, standard care from the staff during their stay in the postpartum ward, without any breastfeeding referral midwifery or nursing. This group of women was selected by systematic random sampling during the two months prior to the intervention.

In addition, to complement the quantitative analysis, a follow-up institutional survey was conducted six months after birth in order to determine the total duration of exclusive breastfeeding, any incidents that occurred with breastfeeding, and the mothers’ level of satisfaction with the care received during their hospital stay. The survey consisted of 13 questions with dichotomous (yes/no) answers, plus one question about satisfaction with breastfeeding during hospitalization, which had 5 possible Likert-type answers. The survey was used as a complementary component to contextualize and interpret the quantitative findings of the study. Responses were compared between the two study periods in order to identify possible differences in the perception of professional support during hospitalization and after discharge, as well as family support and overall satisfaction with the breastfeeding experience during their stay in the unit. The results allow us to interpret possible differences.

### 2.2. Variables

For the study, the following variables were taken into account: maternal age; parity; gestational age (weeks); onset of labor (spontaneous/induced); epidural analgesia; intrapartum antibiotherapy; oxytocin use; episiotomy; type of birth (vaginal/caesarean section); newborn sex (male/female); newborn weight (grams); Apgar score (1–5 min); umbilical cord arterial blood pH; early breastfeeding (first hour); exclusive breastfeeding at discharge; phototherapy in newborn; LATCH scale and bottle-feeding. Survey variables: time of exclusive breastfeeding (months); breastfeeding guidance prior to and after hospital discharge; difficulties breastfeeding in the first few days after discharge; family support with exclusive breastfeeding, and satisfaction about exclusive breastfeeding with hospital staff during hospital admission.

### 2.3. Data Analysis

The data were extracted and fed into an IBM SPSS Statistics 29 program database for analysis. A descriptive analysis was performed for the variables, expressed in terms of counts (*n*) and percentages (%). Quantitative variables were expressed as the mean and standard deviation (SD). An inferential analysis was carried out using the chi-square statistic, Fisher’s exact test, and Student’s *t*-test statistic. In order to control for possible confounding factors, a multivariate analysis was performed using multiple logistic regression, including in the model those variables that were clinically relevant or showed an association in the bivariate analysis. The dependent variable was exclusive breastfeeding success at six months (yes/no). The results were expressed as adjusted odds ratios with 95% confidence intervals. An error α of 5% (*p* ≤ 0.05) was assumed and the exact *p*-values for each statistic were shown.

### 2.4. Ethical Considerations

Data were extracted from the women’s medical records, fully anonymized by authorized personnel, and then fed into the database for further processing and analysis. The study is subject to Organic Law 3/2018 on the Protection of Personal Data, which ensures the anonymity of the data and the confidentiality of the participants, as well as to the standards set out in the Declaration of Helsinki, which governs ethical research involving human subjects. Permission to conduct this research was obtained from the neonatal unit, as well as authorization from the Research Ethics Committee of the Province of Córdoba, SICEIA-2024-001514. Reference: 5941. Date: 3 July 2024. Consent to Participate declaration does not apply, as this is a retrospective study of an institutional intervention.

The authors used Qed Science in order to strengthen the manuscript and improve it. After using this tool/service, the authors reviewed and edited the output as needed and take full responsibility for the content of the publication.

## 3. Results

The total study sample was *N* = 136 women. The mean age of the mothers was 32.2 (SD ± 5.81) years, and the mean weight of the newborns was 3210 (SD ± 380) grams. 49.3% were primiparous women and 50.7% were multiparous; 34.6% of them had undergone induced labour, and 89% had received epidural anesthesia. 86.8% of the total births were vaginal births, and 13.2% were caesarean sections. 95% initiated early breastfeeding within the first hour after birth. 74.3% were exclusively breastfeeding at discharge, and 18.4% were mixed breastfeeding at discharge. Only 6% of the newborns needed phototherapy. Sociodemographic and obstetric data are presented in Table 1.

Baseline characteristics were not significantly different between pre and post intervention (Table 1), and improvements were consistent across multiple early outcomes (Table 2). Data analysis found significant differences between the intervention and control groups in some of the outcome variables. Thus, the percentage of exclusive breastfeeding at discharge was significantly higher in the intervention group than in the control group (86.8% vs. 63.2%; *p* = 0.003). When taking into account the type of delivery, the percentage of exclusive breastfeeding at discharge was higher among women who had had a normal birth compared to those who had had a caesarean section (91.4% vs. 60%; *p* = 0.007). The percentage of mixed breastfeeding at discharge was significantly lower in the intervention group (10.3% vs. 27.9%; *p* = 0.009); also, the percentage of supplementation was lower in the intervention group (23.5% vs. 41.2%; *p* = 0.028). Significant differences between the two groups were found in the treatment with phototherapy, where a lower proportion of newborns needed this treatment in the intervention group than in the control group (1.5% vs. 11.8%, *p* = 0.033), although this figure should be treated with caution given the small number of events. Finally, exclusive breastfeeding rates at 2 months were found to be higher in the intervention group than in the control group (97.8% vs. 83.2%; *p* = 0.031). However, no statistically significant differences were found in the duration of exclusive breastfeeding at 4 or 6 months or in the Latch scale between the two groups.

In addition, the survey data (It should be noted that not all the women responded to the survey: 46 in the intervention group and 46 in the control group responded, which represents 67.6% of each group) showed higher satisfaction rates in the intervention group, with 95.7% of women in the intervention group reporting feeling satisfied or very satisfied with the breastfeeding care they received, compared to only 63% in the control group (*p* < 0.001). In the same way, a significantly higher percentage of mothers in the intervention group reported feeling supported with breastfeeding during their hospital stay compared to mothers in the control group (91.3% vs. 44.4%; *p* < 0.001). In addition, a lower percentage of mothers in the intervention group reported that returning to work was an obstacle to exclusive breastfeeding (30.4% vs. 48.9%; *p* = 0.076), although the differences were not significant. We compared respondents who answered the survey with those who did not in terms of baseline characteristics (Table 1) and reanalyzed the survey-derived outcome measures using inverse probability of response weighting and multiple imputation (Table 2).

In general, as the study design is a retrospective quasi-experimental pre-post design and the baseline characteristics are largely similar (Table 1), the consistency of the improvements in early outcome measures points to a clear association between the results. Data are shown in Table 2.

The multiple logistic regression (MLR) analysis aimed to identify possible confounding factors. After adjustment, two remaining variables were associated with successful exclusive breastfeeding at 6 months. First, the presence of breastfeeding problems during the first few days after discharge was associated with a significant reduction in the probability of continuing exclusive breastfeeding at 6 months. In addition, breastfeeding support after hospital discharge by professionals was associated with a higher probability of success. The model demonstrated modest explanatory power (Nagelkerke R^2^ = 0.173). The Hosmer–Lemeshow goodness-of-fit test was non-significant, χ^2^ = 0.149, *p* = 0.929, indicating adequate model fit. Data are shown in Table 3.

## 4. Discussion

The findings of this study appear to reinforce the importance of interventions and follow-up in achieving better outcomes in breastfeeding. Breastfeeding support availability in hospital via a breastfeeding midwife increased breastfeeding rates in hospital and maternal satisfaction. Thus, it has been found that educational interventions in breastfeeding are effective, as demonstrated by many studies and systematic reviews [12,16]. Another recent systematic review and meta-analysis found that educational interventions in breastfeeding increased the rate of exclusive breastfeeding at 6 weeks, and are even more effective when theory and practice are combined [17]. In another review study comparing educational interventions with the standard education provided in hospital, the authors concluded that there is evidence to support breastfeeding education versus standard hospital information across diverse settings [18]. However, most studies involve various educational interventions and follow-up over time, extending beyond the immediate postpartum period, which helps to increase the duration of exclusive breastfeeding [19]. This is demonstrated in the systematic review and meta-analysis by McFadden et al., who concluded that counselling provided at least four times after birth is more effective than counselling provided only before birth and/or fewer than four times [12]. In this regard, the present study is limited to interventions carried out solely during hospitalization, without providing any follow-up for the mothers over time. This may be the reason why this study did not achieve better results in terms of the duration of exclusive breastfeeding at 6 months. There is a systematic review in which the authors did find a longer duration of exclusive breastfeeding with educational interventions only during hospital admission, although in this case the children required a longer hospital stay than those in our study, who were only admitted for 24 to 48 h [20]. Other studies have shown that more sustained support over time may be linked to higher breastfeeding rates at 6 months, as reflected in the systematic review and meta-analysis by Blackmore et al. [21], which included 19 randomized controlled trials comparing virtual lactation support with other postnatal maternity care; virtual lactation support was associated with increased exclusive breastfeeding rates at 1 month and 6 months postpartum, although, unlike in our study, in this case the support continued after the birth. In this regard, in another recent systematic review, the authors found no differences in exclusive breastfeeding rates at 6 months following continued support from midwives during breastfeeding, although they acknowledged the risk of bias in a large proportion of the studies [22].

This study appears to show a reduced need for phototherapy among babies of mothers in the intervention group, which would suggest a lower risk of jaundice; this could be attributed to the breastfeeding support these mothers receive during their stay in hospital. The term ‘breastfeeding jaundice’ is commonly used to describe neonatal jaundice resulting from suboptimal fluid and caloric intake during the first 7 days of life [23]. Recent studies suggest that, although babies who are exclusively breastfed may be at greater risk of developing jaundice, this is because it takes time for the mother’s milk supply to become constant, rather than being due to the milk itself; therefore, recommendations are aimed at improving breastfeeding rather than discouraging it. In fact, the American Academy of Pediatrics recommends continuing with breastfeeding whilst providing support to ensure sufficient intake [23,24]. Other studies also support the idea that there is no effect of exclusive breastfeeding on the incidence of jaundice in infants aged 0–72 h [25].

Finally, in the present study, women in the intervention group reported higher levels of satisfaction. In this regard, in a study also conducted in Spain involving interventions in breastfeeding during hospitalization, women in the intervention group also reported higher levels of satisfaction [26]. Other studies also report better outcomes in terms of mothers’ satisfaction following an intervention to improve the quality and duration of breastfeeding [27]. However, other authors found no significant differences in satisfaction in their breastfeeding intervention study [28].

### Strengths and Limitations

This study, which uses a complementary descriptive survey to strengthen the findings, has certain limitations. Firstly, the duration of the intervention was limited to two months, meaning that the sample size was also limited. Furthermore, the study was conducted in a single hospital, and it would be worthwhile to carry out a multicentre study that would also allow for a larger sample size. Moreover, the retrospective, non-randomized design, as well as the outcome measures and post-discharge exposures, is subject to recall bias and non-response bias. Given the retrospective pre-post design without a contemporaneous control, early outcome comparisons should be interpreted as associations rather than causal effects. Finally, the interventions in this study were limited to the postpartum hospital stay; therefore, it would be interesting to carry them out and conduct follow-up during the first six months to improve exclusive breastfeeding rates.

The main strengths of this study lie in its complementary descriptive survey, which successfully contextualizes quantitative medical data with the mothers’ actual perceptions from a 6-month survey. Additionally, the strong baseline homogeneity between the study groups minimizes confounding variables, while the use of advanced statistical techniques—such as inverse probability of response weighting and multiple imputation—robustly mitigates non-response bias from missing survey data.

## 5. Conclusions

This study suggests that implementing a dedicated, breastfeeding-specialist midwife in postnatal units could be a highly effective institutional strategy for optimising early outcomes and improving the quality of care. Having a professional focused exclusively on breastfeeding support could significantly increase rates of exclusive breastfeeding during hospitalisation, reduce infant morbidity and improve maternal satisfaction. However, the lack of sustained effects at six months highlights that in-hospital interventions alone are insufficient for long-term success; thus, continuous post-discharge support is essential to enable mothers to achieve the goal of exclusive breastfeeding up to six months.

## Figures and Tables

**Table 1:** Sociodemographic and obstetric variables of women (*N* = 136).

	Intervention *N* = 68		Control *N* = 68		
Variable	*n*	%	*n*	%	*p*
Age (years) *	32.3 (SD ± 5.44)		32.2 (SD ± 6.19)		NS
Parity					
Primiparous	36	52.9	31	45.6	NS
Multiparous	32	47.1	37	54.4	
Epidural analgesia	58	85.3	63	92.6	NS
Onset of labour					
Spontaneous	47	69.1	42	61.8	
Induced	21	30.9	26	38.2	
Intrapartum antibiotherapy	12	18.2	16	23.5	NS
Oxytocin use	31	45.6	32	47.1	NS
Type of birth					
Spontaneous	53	77.9	52	76.5	NS
Instrumented	5	7.4	8	11.8	
Caesarean section	10	14.7	8	11.8	
Episiotomy	11	16.2	10	14.7	NS
Newborn sex					
Male	40	58.8	31	45.6	NS
Female	28	41.2	37	54.4	
Skin-to-skin contact	65	95.6	59	86.8	NS
Early breastfeeding	66	97.1	63	92.6	NS

* Data are mean (SD); NS: not significant.

**Table 2:** Results and differences between two groups as regards breastfeeding (*N* = 136).

	Intervention *N* = 68		Control *N* = 68		
Variable	*n*	%	*n*	%	*p*
Exclusive breastfeeding at discharge					
Yes	59	86.8	43	63.2	0.003
No	9	13.2	25	36.8	
Mixed breastfeeding at discharge					
Yes	7	10.3	19	27.9	0.009
No	61	89.7	49	72.1	
Supplementation in hospital					
Yes	16	23.5	28	41.2	0.028
No	52	76.5	40	58.8	
Phototherapy in newborn					
Yes	1	1.5	8	11.8	0.033
No	67	98.5	60	88.2	
Latch Scale					
Satisfactory	44	69.8	45	67.2	0.743
Unsatisfactory	19	30.2	22	32.8	
Duration of exclusive breastfeeding up to 6 months *^/^**	5.00 (1.50)		4.63 (2.23)		0.375
At 2 months **	45	97.8	40	83.3	0.031
At 4 months **	35	76.1	35	72.9	0.725
At 6 months **	30	65.2	29	59.2	0.540
Satisfaction with exclusive breastfeeding during admission					
Satisfied or very satisfied **	44	95.7	29	63	<0.001
Not satisfied **	2	4.3	17	37	

* Data are mean (SD). ** Data on duration of exclusive breastfeeding up to 6 months and satisfaction (*n* = 46 in each group).

**Table 3:** Shows the results of MLR model.

Variable	Adjusted OR	CI 95%	*p*
Age (years)	0.96	0.86–1.06	NS
Parity	1.52	0.75–3.06	NS
Type of birth (vaginal/caesarean)	0.73	0.18–2.90	NS
Early breastfeeding	0.79	0.11–5.37	NS
Antibiotic prophylaxis	0.73	0.21–2.51	NS
Initial breastfeeding problems	0.28	0.10–0.74	0.011
Breastfeeding support after discharge	3.06	1.16–8.05	0.023
Phototherapy	2.58	0.35–18.60	NS

Nagelkerke’s R^2^: 0.173; Hosmer–Lemeshow test: χ^2^ = 0.146; *p* = 0.929. NS: not significant.

## Data Availability

The datasets generated and analyzed during the current study are not publicly available due to ethical restrictions protecting participant confidentiality but are available from the corresponding author upon reasonable request.

## Abbreviations

The following abbreviations are used in this manuscript:

EsdE	Spanish Health Survey
SD	Standard deviation
SICEIA	Information System for Research Ethics Committees in Andalusia
UNICEF	United Nations Children’s Fund
WHO	World Health Organization
